# Supplementary material for: Hearing screening beyond the clinic: Childhood cancer survivors’ perspectives on a novel hearing screening program
Source: BMC Health Serv Res. 2026 Apr 11;26:724. doi: 10.1186/s12913-026-14491-5 (PMC13196202; doi:10.1186/s12913-026-14491-5)
Supplement: Supplementary file 1 — Supplementary Material 1 [file 12913_2026_14491_MOESM1_ESM.docx]

### Supplementary TABLE S1: Semistructured interview guide (translated from German to English).

Note: The interview used in our study was developed for this study and has not previously been published elsewhere. Main questions were further explored by follow-up questions.

| Introduction | Interviewer introduced herself briefly and explained again the aims of the HEAR-study and the procedures of the interview. |
| --- | --- |
| Introductory questions | Why don't you start by telling me what went through your mind when you received the invitation letter for our study?   - Why did you decide to take part in the study? - What expectations did you have of the study?   What do you think about research?   - Have you already participated in other studies? - Would you like to be involved in the development of research projects/research questions? |
| Previous hearing tests | And then you decided to do a hearing test for the study. Have you already had any kind of hearing test before? (e.g. military service, university, education, follow-up care)  If yes: Tell me about this hearing test.   - Who ordered the hearing test? Why? - When was it? (After completion of therapy?) - Where did the hearing test take place? - How did you make the appointment? - What was the hearing test like? - What was the result? - How do you remember the hearing test? - How much effort was the test for you? |
| Hearing problems | If hearing problems are known: What is it like for you to live with hearing problems?   - What is it like to know that you have hearing problems? - Does the issue come up often? - When do you notice it most? - What measures have been taken? |
| Hearing test at the hearing aid shop | Now I would like to know about your experience with the hearing test at the hearing aid shop.  Tell me about the hearing test at hearing aid shop.   - How come you made the appointment there? - How long did you have to wait for an appointment? - How did you travel to the branch? How long? - How much effort was involved for you? - What was the hearing test like? - What information did you receive from the acoustician? - How did you feel during the test? - Did you think about the possible test result? - How was it for you to talk to the acoustician about your hearing compared to a doctor? |
| After the hearing test | How did you feel after the hearing test?  How did you react when you received the result of your hearing test?   - How were you informed of the result? - How did you feel about the result afterwards?   Did the hearing test change anything in your everyday life?   - Did/will you discuss the result with someone?   - If yes: With whom?   - If yes: What did they do/say?   - If no: Do you still plan to do this? |
| Advantages & disadvantages of hearing test options | If you now compare this and your previous hearing test experience (if applicable): What are the differences between the two tests?   - What are the advantages/disadvantages of both?   What are the differences between an acoustician in the hearing aid shop and a doctor who carries out the hearing test?   - What are the advantages/disadvantages on both sides?   If you had to have a hearing test regularly, what would be most important to you during the visit?  Where would you go for a hearing test in the future?   - Why there? - What are the advantages for you personally of doing it at this location?   What are the disadvantages for you personally if you do it somewhere else? |
| Follow-up care setting | I would now like to talk to you about follow-up care after a cancer diagnosis. By this I mean examinations, such as blood tests, to detect any effects of your treatment or illness at an early stage after treatment has been completed.  Do you go for a follow-up examination or another regular check-up? (e.g. annual visit to the general practitioner)  What do they examine? |
| Transition to adult follow-up care | What was the transition from pediatric to adult follow-up care like for you?  Can you remember the information you received about follow-up care?   - What was particularly emphasized? - Who did you receive the information from? - Was the information verbal/written? - Would you have liked more information at the time? - Was hearing follow-up a topic? |
| Current follow-up care | What do you think about follow-up care today?   - What is the current importance of follow-up care in your life?   - Do you still go to follow-up examinations?     - Where?     - Why / why not?     - How does it work?     - Do they check your hearing?     - How does it make you feel?   What could be improved in follow-up care?   - What would you wish for new survivors? - What is already going well in terms of follow-up care? |
| End | Has your view of follow-up care changed as a result of the study?  What expectations did the study (not) fulfill?  What do you take away from this study?  Now I've almost finished my questions. Perhaps you would like to tell me something else that I haven't asked?  Do you have any questions for me or about the study? |

### Supplementary TABLE S2: Quotes in original language (German or French) and English translations.

| **Nr.** | **Participant** | **English translation** | **Original German/French quote** |
| --- | --- | --- | --- |
| **1** | 29-year-old-woman | I found it very straightforward and very quick. I went into the room and after ten minutes or so I was out again. | Also ich fand, es war sehr unkompliziert und sehr schnell. Also ich ging dort in diesen Raum und, ich glaube, nach, ich weiss auch nicht, zehn Minuten oder so war ich wieder draussen. |
| **2** | 35-year-old woman | If you're having a follow-up check-up with the oncologist anyway, for example, and you can do it all at the same time: a lab appointment, an X-ray appointment and a hearing test at the same place on the same date, most people would certainly appreciate that. […] Then you don't have to drive to this place, that place and yet another place. You could do it all in one go. | Wenn man eh in einer Nachkontrolle ist beim Onkologen zum Beispiel und man kann das gerade im gleichen Zirkus machen, indem man sagt, ja man hat noch schnell den Labortermin hier, noch schnell den Röntgentermin dort und man kann auch noch gerade am gleichen Ort diesen Hörtest gerade am gleichen Termin abspulen, ist das sicher für die meisten Leute dankbar. […] Dann musst du nicht einmal hierhin fahren, einmal dorthin und einmal dahin. Dann könntest du alles in einmal machen. |
| **3** | 39-year-old man | I was warmly welcomed and taken care of. They also told me a bit about what they were doing and why, and explained how the hearing [physiology] works. They also explained possible hearing impairments [...] and how the test is conducted. I really felt very comfortable there and if I had had any questions, I would certainly have been able to ask them, but they were actually answered by the person during the process | Ich wurde freundlich aufgenommen, man hat sich um mich gekümmert, man hat mir auch ein wenig erzählt, was man jetzt alles macht und warum man das macht, man hat auch den ganzen Gehörvorgang nochmals erklärt. Und auch was die möglichen Beeinträchtigungen sind, die passieren könnten und wo die passieren und wieso und wie der ganze Test abläuft. Also ich habe mich dort wirklich sehr wohl aufgenommen gefühlt und wenn ich Fragen gehabt hätte, hätte ich diese sicher auch stellen können, aber die wurden durch die Person eigentlich schon beantwortet im Verlauf. |
| **4** | 39-year-old woman | A hearing aid shop is low threshold. It's not: ‘Ah, I'm going back to the hospital now’. Depending on the situation, you might not have the best associations with hospitals. And if you're just like anyone else who goes there for counseling, it's actually pleasant. It's not the feeling of being a patient, but simply a customer. | Ich denke so… eben in einem Hörgeschäft ist es natürlich sehr so… es ist so niederschwellig. Es ist nicht: «Ah, ich gehe jetzt wieder ins Spital.» und so. Je nachdem hat man vielleicht auch nicht die besten Assoziationen so mit Spital und allem. Und dann ist es sicher so in diesem Rahmen, wie wenn man einfach jemand wäre, der sich sonst beraten lässt dort, ist auch noch angenehm. Es ist nicht so das Gefühl Patientin, sondern einfach Kundin. |
| **5** | 45-year-old woman | It's a place where I can go again at any time. Let's say I want to know something more, or I still want to have this photo [otoscopy]. I can just write an email or call. The shop is always available. I mean these specialists [doctors], just getting an appointment at all... they are somehow so far away. Not so close. Not so easy to reach. And that seems to me to be a clear advantage [of the hearing aid shop] | Es ist ein Ort, wo ich jederzeit wieder hinkann. Sagen wir, ich möchte jetzt doch noch etwas wissen oder sagen wir, ich möchte dieses Foto jetzt doch noch haben. Ja dann kann ich einfach ein E-Mail schreiben oder anrufen oder so. Es ist jemand, der irgendwie wie immer erreichbar ist. Und dann, ich meine gerade diese Spezialisten [redet von Ärzten und Ärztinnen], nur schon, dass man überhaupt einen Termin bekommt… ja sie sind irgendwie so weit weg. Nicht so nah. Nicht so einfach erreichbar. Und das scheint mir schon ein deutlicher Vorteil zu sein. |
| **6** | 41-year-old man | I see a disadvantage [of the hearing aid shop] for someone who might be recovering and who still has visible sequelae of the illness [later he mentioned hair loss as an example]. That may really hold someone back from going to a public place. | Euh, je vois un désavantage pour quelqu’un qui serait peut-être en rémission, qui a encore des, qui a encore des séquelles visibles de la maladie. Euh, ça peut effectivement retenir, euh, d’aller dans un espace public quoi. |
| **7** | 19-year-old man | [Examinations in hearing aid stores] have no medical components. They don't have a doctor to interpret [the results]. Not to question the competence of a hearing aid acoustician, but they don't have medical staff who know my diagnosis and can reliably assess [the hearing]. And that's why I would say the hearing aid shop is certainly quicker and better for people who just want a rough assessment of how well they can hear. But it's not suitable for people who really need medical follow-up treatment and follow-up checks like I do, because they don't meet all the requirements. Even if it might be a bit more practical | Auf der anderen Seite ist, hat es auch keine medizinische Komponenten. Also es hat nicht einen Arzt, der das auswertet. Also gar nicht, um die Kompetenz eines Hörgeräteakustikers in Frage zu stellen, aber es hat halt kein medizinisches Personal, das auch meine Diagnose kennt und einschätzen kann, zuverlässig. Und deshalb würde ich sagen Amplifon ist sicher schneller und besser für Leute, die einfach mal eine grobe Einschätzung möchten, wie gut sie hören. Aber für Leute, die jetzt wirklich so wie ich in medizinische Nachbehandlung müssen und in Nachkontrolle gehen, nicht geeignet, weil es die Anforderungen nicht erfüllt. Auch wenn es vielleicht ein bisschen praktischer wäre. |
| **8** | 39-year-old woman | If I had to do such tests more often, the most decisive factor would probably be where it is easiest to access... Locally. So it would be easy to integrate into everyday life. I wouldn't care whether it was a clinic or a hearing aid shop. Where I live, I would probably rather go to the [hospital name]. On the other hand, you wait longer in the clinic. At [hearing aid shop name], they "timed" it nicely that it was just my turn. That is a further aspect. So above all: what is easiest to integrate into everyday life? | Also wenn es häufiger wäre [regelmässige Hörtests], wäre wahrscheinlich am ausschlaggebendsten, wo es einfach am einfachsten zugänglich ist. Also örtlich. Dass es am einfachsten zu integrieren wäre in den Alltag. Daher… mir wäre es, glaube ich, wirklich egal, ob es Klinik wäre oder eben zum Beispiel ein Hörgeräteshop, obwohl jetzt vom aktuellen Setting her vom Wohnen fast lieber ins (Spitalname) oder so. Obwohl manchmal in der Klinik wartet man dafür länger. Bei Amplifon haben sie es jetzt schön «getimed», dass ich gerade drankam. Daher wäre das auch noch wieder ein Aspekt. Also vor allem, was lässt sich am einfachsten integrieren halt in den Alltag. |
